# The Relation between Social Capital and Academic Motivation of Students: A Study of Health Professional Education in Japan

**DOI:** 10.3390/ejihpe11010011

**Published:** 2021-02-09

**Authors:** Yuka Koyanagi, Myo Nyein Aung, Motoyuki Yuasa, Miwa Sekine, Okada Takao

**Affiliations:** 1 Graduate School of Medicine, Juntendo University, Tokyo 113-8424, Japan; y-koyanagi@juntendo.ac.jp; 2 Tokyo Ariake University of Medical and Health Sciences, 2-9-1 Ariake, Koto-ku, Tokyo 135-0063, Japan; 3 Juntendo Advanced Research Institute for Health Sciences and Faculty of International Liberal Arts, Juntendo University, 2-1-1 Hongo, Bunkyo-ku, Tokyo 113-8421, Japan; 4 Department of Public Health, Graduate School of Medicine and Faculty of International Liberal Arts, Juntendo University, Tokyo 113-8421, Japan; moyuasa@juntendo.ac.jp; 5 Division of Medical Education, Graduate School of Medicine, Juntendo University, Tokyo 113-8421, Japan; miwa@juntendo.ac.jp (M.S.); tokada@juntendo.ac.jp (O.T.)

**Keywords:** motivation, medical education, health profession education, social bonds, social motivation

## Abstract

Academic motivation consists of reward-based extrinsic motivation and curiosity-based intrinsic motivation. Students studying at university or college develop several new social connections with friends, classmates, and teachers, in addition to their family and community. Belonging to their networks, students acquire opinions, appreciation, trust, and norms of the society. Whether those social connections enhance the motivation of university students for academic work is a question yet to be answered in the context of health profession education in Japan. Judo-therapist education is a form of health profession education in Japan. This study aimed to measure the academic motivation and social capital (SC) of judo-therapist students in Japan, and to find the relation between social capital and academic motivation. This cross-sectional study recruited a total of 2247 students applying multi-stage sampling across Japan. A Japanese version Academic Motivation Scale (AMS) measured the learning motivation in three constructs: (1) intrinsic motivation (IM); (2) extrinsic motivation (EM); and (3) amotivation (alpha 0.94). A newly-developed 46-itemed, 4-pointed scale measured social capital (SC) in five constructs: (1) family relations, (2) on-campus friends, (3) off-campus friends, (4) classroom social capital; and (5) regional social capital (alpha 0.85). Robust regression analysis treated all constructs of SC as independent variables and IM and EM as dependent variables respectively in the three models. Among the average level of constructs, the family SC average level was the highest. Classroom SC was less than family SC and community SC was the lowest. Intrinsic motivation is positively influenced by classroom SC the most, followed by family SC, on-campus friends’ SC, and community SC. Extrinsic motivation is positively influenced by classroom SC the most, followed by family SC, on-campus friends’ SC, and community SC. Amotivation is negatively influenced by social capital constructs except external friends’ SC. In conclusion, social connections have the power to enhance the motivation of university students’ academic work within health profession education. The relations, trust and bonds developed in the classroom may allow an adult learner’s motivation to evolve into autonomous intrinsic motivation and prevent amotivation.

## 1. Introduction

A strong motivation for learning is the driving force behind students’ academic achievement. Such academic motivation was categorized by Vallerand into “intrinsic motivation”, “extrinsic motivation”, and “amotivation”, based on self-determination theory (SDT) [1,2,3]. Academic learning may be categorized in terms of the cognitive domain, psychomotor domain and affective domain, with academic motivation pertaining to the affective domain [4].

Contemporary research in medical education and health profession education increasingly focuses on the individual and social factors influencing students’ motivation for academic work [4]. In his qualitative research, however, Harvard sociologist Putnam said that the connection within a community and within family could have a profound impact on the education of young people [5]. He also discussed the impact of social changes and family relations on the education of children and youth in his book “Our kids, shattered American dreams” [5]. Twenty-first century social changes have also occurred in Japan, where harmony, trust and respect have been culturally-relayed social values. Whether the power of social relations, bonds and trust, collectively termed “social capital”, has an influence on adult learners’ academic motivation in Japan, is yet to be studied.

### 1.1. Academic Motivation

Academic motivation has been an area of extensive research applying several different theories. The globally dominant theory, which is also most relevant to education is SDT [6]. There is a spectrum of motivation constructs theorized in SDT from amotivation, reward-based extrinsic motivation to final autonomous intrinsic motivation. Reward-based extrinsic motivation consists of three sub-constructs: (1) extrinsic motivation external regulation (the energy to try and attain rewards, such as a better academic grades, a degree, or a higher income); (2) extrinsic motivation-identified regulation (the motivation resulting from external factors such as personal goals, institutional goals, or national goals); (3) extrinsic motivation-introjected regulation (the motivation resulting from the desire to verify one’s capability to achieve rewards). Regarding academic work, reward-based motivation is short-lived, accompanied with exhaustion and anxiety and can lead to an unhealthy response to academic stress [7].

Intrinsic motivation is developed from internationalization of those reward-based motivations to a more independent form of motivation [8]. The intrinsic motivation constructs for academic work are: (1) intrinsic motivation to know (the eagerness to gain new knowledge); (2) intrinsic motivation toward accomplishment (the pleasure in completing tasks); (3) intrinsic motivation to experience stimulation (the inspiration to learn new things becomes a state of love to learn). Amotivation is lack of intentionality in doing certain behavior such as academic work.

A series of SDT research in the 21st century increasingly focused on the influences of proximal social contexts—families, peer groups, schools, teams, and work organizations—on the individual’s motivation. According to SDT, students are motived for their academic work in an environment favoring “autonomy, competence and relatedness”. Pedagogical research in existing literature highlighted autonomy-favored classrooms and school environment. However, learning styles of the students can be different across cultures. Japanese students have a more reflective learning style as the cultural value in Japan is high in both group collectivism and institutional collectivism, and uncertainty avoidance [9]. In this context, contribution for the good of people and community, teachers’ and peers’ relationship, and sense of belonging to a particular society may motivate students.

### 1.2. Social Capital

Meanwhile, such a phenomenon, the capital, emerged from a supportive social network is being referred to as social capital [10]. Social capital (SC) is a social resource resulting from how well people in the community are connected through their network, trust, and norms of the society [11]. Just as neighborhood and residential areas are important in the community for residents, the university campus, the classroom and relationships with friends and teachers on the campus are important for students who spend most of their time at university [12]. Several researches in the higher education setting reported the role of social capital for academic achievement, mostly in qualitative studies [13]. Although the idea of social capital and its implications are very diverse, social capital is tangible. Measurement of social capital is very contextual. Individually, it can be measured applying its construct as variables [14,15]. Research quantifying social capital individually, in the higher education setting, particularly Judo-therapy education and relating it to affective domain in education, as well as motivation, [4] is still limited and not yet conducted in Japan.

Previous studies reported that people living in well-connected and bonded communities usually have better health and well-being than those living without strong social cohesion [5]. Moreover, children from such communities progress very well in education. According to Haga and Sakamoto, trust, affinity, network, and reciprocity are components of school SC in campus life [16]. Anderson also revealed that teacher SC and classroom SC are related to achievement in primary education [17]. Furthermore, previous studies conducted in Japan have investigated the social capital influence on primary schools and high school students’ motivation. However, no past study has investigated such a relation amongst university students especially those who are in health profession education. 

### 1.3. Judo-Therapy Education Setting

Judo-therapy is a medical therapy used in Japan [18]. It is of Japanese origin and has evolved as a scientific health practice, traditionally relayed over the past 1300 years [19]. Judo-therapy education is a well-established health profession education carried out in academic institutions within every prefecture of Japan. There are currently 16 universities in Japan providing a 4-year university degree, and 93 institutes providing a 3-year technical diploma.

Students learning judo-therapy study basic sciences, clinical subjects and internship in order to get a degree or diploma. All graduates then have to pass the national license examination to obtain a licensure to practice under national health insurance [20]. Judo-therapists serve to treat acute trauma and injury with non-operative procedures [20]. They also serve to assist the orthopedic department in many hospitals [21]. In the communities, judo-therapy clinics serve as the clinic for common, acute and minor musculo-skeletal problems and health promotion relating to exercise and rehabilitation.

Enrolling into judo-therapy classes can be the students’ own choice or their parents’ choice. After entrance to the school, students will experience campus life, class activities, and extracurricular activities, as well as ongoing communication with their family, resulting in new social contacts and social interaction. Therefore, these social experiences may have an influence on students’ motivation for their academic work. Moreover, drop-outs and failures among university students nowadays are increasingly reported as being associated with broken families, children of single mothers, and other such social problems [22,23].

In the light of observation and evidence discussed above, we set the hypothesis to determine if the different levels of social capital belonging to the students is pertaining to the levels of academic motivation such as intrinsic motivation, extrinsic motivation and amotivation, and to determine the direction of association with statistical analysis. To execute it, first, we carefully measured the level of academic motivation constructs and social capital constructs in a representative sample.

The present study sought to investigate further the relation between social capital and human capital, especially though the motivation of students in higher education. Therefore, this study aimed (1) to measure the academic motivation of judo-therapist students in Japan; (2) to measure the social capital of judo-therapist students in Japan; and (3) to find the relation between social capital and academic motivation.

## 2. Method

This was a cross-sectional study. Surveys at 20 schools applied self-administered, structured questionnaires to collect the data. Data collection lasted from December 2018 to April 2019. Nationwide data collection recruited a total of 2248 students across Japan applying multi-stage sampling. Sample size was estimated using Yamane formula to represent the target population of 26,287 students studying judo-therapy across Japan, inflation for multistage design, compensation for the non-responding and enough power for Robust multivariable regression analysis. The sampling frame included all the universities and colleges teaching judo-therapy classes in Japan where stratification considered three criteria: (1) type of schools: university and colleges; (2) grades within each school and (3) geographical areas. Within each region, one university and one college were randomly selected, in the six out of eight regions of Japan. Within each school, all students from each grade were invited to participate in the research. Altogether, 20 schools were selected to include technical colleges and universities within the different provinces of Japan.

Measurement of students’ motivation applied the Academic Motivation Scale (AMS) [3]. It is based on SDT [1]. It is a 28-itemed, 7-pointed scale and is freely available for research [24]. The AMS has been applied in medical education and health profession education research in many countries [25]. In this study, a transculturally-translated Japanese version of the AMS (Cronbach α 0.94) was applied to measure students’ motivation in three constructs: (1) intrinsic motivation (IM); (2) extrinsic motivation (EM); and (3) amotivation (AM). Transcultural translation followed the following steps: forward translation, backward translation, pilot test for readability, comprehension and reliability [26]. IM score ranged from 12 to 84. EM score also ranged from 12 to 84. AM score ranged from 4 to 28.

Measurement of social capital (SC) applied a 46-itemed, 4-pointed scale in five constructs: (1) family social capital; (2) on-campus friends’ social capital; (3) off-campus friends’ social capital; (4) classroom social capital; and (5) community social capital (Cronbach α 0.85). The scale, used in previous research among primary school students, was adapted to fit the university student population of the current study [15]. Item-objective congruence by three experts was performed to verify content validity. A pilot study proceeded actual survey, to test reliability, comprehension and readability. The adapted instrument is provided in the Appendix in English. Significant social relations which belonged to university students studying judo-therapy in the Japanese context such as family bonding, friends on the campus, friends off the campus, relations within the classroom, and relations with community members were carefully analyzed to measure social capital.

STATA analytical software version 15 (Special Edition, Stata Corp., 4905 Lakeway Drive, College Station, TX, USA) was applied for data analysis. Descriptive analysis was applied to report characteristics of the sample, the level of academic motivation and social capital (Table 1 and Table 2). Mean and standard deviation (SD) for the dependent and independent variables are provided for summary. The distribution of observed variables was not normal except classroom social capital (Table 2). Therefore, median and interquartile range (IQR) were also presented in the result. To assess the association between dependent and independent variables, robust regression analysis was applied in STATA. Robust multivariable regression analysis treated all 5 constructs of social capital as independent variables and IM, EM and AM as dependent variables respectively in the 3 models. Final models adjusted covariates such as self-made career decision, exposure to the judo-therapy as a patient before enrolling in the university, and work exposure before enrolling in the university. *p*-value < 0.05 was defined as statistical significance with a 95% confidence interval (CI) (Table 3).

The sample size was calculated to have representative sample resulting in sufficient power for the objectives of the research. This was a sample size sufficient for 80% power and accuracy ±1.5% and 95% confidence interval. Statistical significance was determined as *p*-value < 0.05. The power of the sample size was enough to perform robust regression analysis of AMS and SC relationships. In addition, the sample size was sufficient to compensate for non-responses. However, of the 2248 students invited and participated in the study, a total of 2247 (99.95%) completely responded to the AMS questions. We checked the power sample calculation backward. Final sample size is sufficient to represent the population of 26,287 and to have power for more than 80% for the analysis results.

The study obtained ethical approval from the Juntendo University research ethics committee (Jundai Medical no. 2018160). Before data collection at each study site, the researcher explained to the students that participation in the research would have nothing to do with their academic grade. Students could choose freely whether to participate in the study. Informed and consented participants responded to the survey by answering self-administered questionnaires, taking 40 min. Overall, the response rate was 100%.

## 3. Results

A total of 2248 students responded to the study. The proportion of male students (71.24%) was more than female students (28.76%). There were two main types of schools: (1) universities and (2) colleges. More than half of the students (58.85%) belonged to colleges. First-year students comprised 32.22%, second-year students 29.86%, third-year students 27.33%, and fourth-year students 10.59%. 

To determine the economic situation of the students, we asked if they had obtained a scholarship. Almost half of the students, 1110 (49.62%), were recipients of scholarships.

The students were asked about the level of education of their parents to investigate the possible link between the student’s willingness to learn and the level of education of their parents. Of the mothers, 965 were high school graduates (44.93%), 703 were college graduates (32.73%), 426 graduated from vocational schools (19.38%), and 54 were junior high school graduates (2.51%). Of the fathers, 918 were high school graduates (43.82%), 791 were college graduates (37.76%), 293 graduated from vocational schools (13.99%), and 93 were junior high school graduates (4.44%).

Making one’s own decision about career choice is an important factor in determining a student’s academic motivation. Thus, participants were asked whether they had made their own decision regarding their career choice. The majority of students (96.18%) responded that they had made their own career choice to enroll in judo-therapy classes (Table 1). Having a role model for an educational career is important for motivation. There were 1424 students (63.37%) who said they had role models (Table 1).

### 3.1. Academic Motivation Level and Its Constructs: Intrinsic Motivation (IM) and Extrinsic Motivation (EM)

The response rate for three main constructs of AMS—intrinsic motivation (IM), extrinsic motivation (EM) and amotivation (AM) was 100%. The average level of EM was 54.9 ± 14.1 (mean and SD). EM was composed of three constructs. Their average levels (mean and SD) were as follows: EM identification 19.76 ± 5.05; EM introjection 16.13 ± 5.64; and EM regulation 18.99 ± 5.41.

The average level of IM was 54.01 ± 14 (mean and SD). The average levels of IM constructs were as follows: IM to know 20.60 ± 5.30; IM to accomplish 16.83 ± 5.28; and IM to experience 16.58 ± 5.

### 3.2. Social Capital (SC) of Judo-Therapy Students

Overall, the response rate to SC was 99%. The level of family SC was 3.1 ± 0.5 (mean, SD); on-campus friends’ SC was 3.0 ± 0.6; off-campus friends’ SC was 3.0 ± 0.5; classroom SC was 2.7 ± 0.5; and community SC was 2.3 ±0.6. Among the average level of constructs, the family SC average level was the highest. Classroom SC was less than family SC and community SC was the lowest.

### 3.3. Robust Regression Analysis Result

Table 3 shows robust regression analysis of AMS as dependent variable and SC as independent variable.

Model 1 reveals the factors significantly related to intrinsic motivation investigated in robust regression analysis. Intrinsic motivation is positively influenced by social capital constructs—classroom SC the most, followed by family SC, on-campus friends’ SC, and community SC. Social connection in the classroom has the strongest relation to IM of the students.

Model 2 reveals the factors significantly related to extrinsic motivation investigated using robust regression analysis. Extrinsic motivation is positively influenced by classroom SC the most, followed by family SC, on-campus friends’ SC, and community SC. Again, social connection in the classroom has the strongest relation to EM of the students.

Model 3 reveals the factors significantly related to amotivation investigated using robust regression analysis. Amotivation is negatively influenced by social capital constructs with the exception of external friends’ SC. Again, social connection in the classroom is the strongest preventive factor for amotivation.

## 4. Discussion

Motivated students can steadily pass years of academic milestones in college or university, but some students fail to accomplish their goals and leave the school. Several studies had reported the source of motivation such as role model and self-determination. Whether social bonds belonging to students, their representation in the class or a society, can empower students’ academic motivation, is a very interesting research question for the faculties and educators but, it has not been answered by any study yet [13]. (The same question is applied to students studying judo-therapist education.

Earlier in the 20th century, the influence of family relationship and connection in the community were reported to have impact on a youth’s education, by the studies in the United States [10]. In the 21st century, evidence in experimental social psychology signaled that social belonging can empower an individual’s motivation within a particular domain, in a well-known study called “mere belonging” [27]. However, no study has addressed whether bonding in the family, belonging to society or class, or relation in the community, can foster university students’ motivation for academic work, an affective domain in education. The present study, conducted in the contemporary setting of Japan, confirmed such a relation between two concepts, by proving that university students’ motivation for studying the health practice of judo-therapy is influenced by social capital.

In this study, the academic motivation of university students studying judo-therapy in a nationally representative sample of 2247 was measured (Table 2). Characters of judo- therapy students in the sample is similar to annual students’ registry statistics (Table 1). This study reported the first nationally representative measurement result of academic motivation in three constructs: intrinsic motivation, extrinsic motivation and amotivation, following SDT and social capital in five constructs: family SC, on-campus friends’ SC, community SC, classroom SC and off-campus friends’ SC, belonging to judo-therapy students in Japan.

It is the first study of its kind reporting on academic motivation within health professional education in Japan, applying an SDT-based Academic Motivation Scale (AMS). Since Ryan and Deci developed SDT, several research studies have investigated the balance between intrinsic and extrinsic motivation for academic work [2]. Extrinsic motivation constructs measured the reward-based motivation which is important to accomplish tasks for study and grade achievement of the students [3]. Meanwhile, intrinsic motivation constructs reveal curiosity-based, persistent motivation which is the basis for life-long learning. In this study sample, the level of extrinsic motivation was shown to be slightly higher than that of intrinsic motivation (Table 2). Such distribution of constructs is similar to the findings of previous studies conducted in the Asian setting [28,29]. One previous study of medical students reported that such a pattern of higher extrinsic motivation is common among Asian students [30]. This pattern of motivation can lead to anxiety in responding to academic stress. Subsequent discussion will explain how the finding of this study may pave the way to make student motivation more intrinsic.

In addition, the social capital of the college and university students studying judo-therapy in this representative sample was measured. Many previous studies in education research have reported on the social capital of students in primary schools and high schools in Japan. The current study is the first one to investigate the social capital among adult students in higher education—those in college and university studying judo-therapy as a type of health professional education. This study confirmed the relation between social capital and affective domain in education, i.e., motivation for academic work.

Furthermore, the result of the current study (Table 3) showed a statistically significant association between social capital constructs and academic motivation constructs. Intrinsic motivation is positively influenced by social capital constructs—classroom SC the most, followed by family SC, on-campus friends’ SC, and community SC, whereas extrinsic motivation is positively influenced by classroom SC the most, followed by family SC, on-campus friends’ SC, and community SC. Amotivation is negatively influenced by social capital constructs with the exception of external friends’ SC. Therefore, social bonding has the power to motivate students for their academic work (Table 3). Moreover, social capital in the classroom has the strongest influence on students’ motivation, shifting towards intrinsic motivation for academic work.

Classroom SC revealed the strongest relation to intrinsic motivation (β 0.73, 95% CI 0.61–0.96, *p* < 0.001) and extrinsic motivation (β 0.50, 95% CI 0.37–0.63, *p* < 0.001) (Table 3). Comparing those two relations, intrinsic motivation is more relevant to classroom SC. Therefore, classroom SC may cultivate both reward-based and curiosity-based motivation, with a positive shift to intrinsic motivation. A wealth of bonding, trust and relationship-building with classmates and teachers can lead to an increase in students’ motivation, resulting in active and life-long learners. Measurement of classroom SC in current study focused on the teacher and student relationship (Supplementary Material: Social capital scale). Similarly, a systematic review of 43 published studies reported the paramount influence of teacher-student relationships upon student engagement, after adjusting covariates such as family and friends [31].

Off-campus friends’ SC was associated with non-motivation whereas classroom SC revealed a preventive effect on amotivation (β −0.30, 95% CI −0.35–0.24, *p* < 0.001) (Table 3). Interventions targeting classroom SC may help shift away from the current pattern of reward-oriented motivation among judo-therapy students towards more autonomous, curiosity-based motivation.

Other independent variables which were statistically significant in the robust multivariable analysis include the existence of a role model, the self-decision to enter the university, and prior exposure to work before attending university (Table 1 and Table 3). Prior work exposure reflects a slightly older age and maturity of students [32]. Self-decisions highlight autonomy whereas the influence of a role model contributes to self-efficacy [25]. Several psychological studies have reported such factors influencing motivation for academic work. These factors relate to individual perception whereas social capital is the positive supportive power from networks which cultivates trust and bonding among peers or members.

Robert Putnum reported the association of social capital to education amongst American children living in different states [10]. He mentioned other influences such as parental education level, poverty rate, and family structure. He highlighted the significance of social capital as providing positive social support through networking, trust, and social bonding. Similarly, the significance of social capital in the presence of other significant factors can be explained in the current study (Table 3).

The importance of social context in the interest and motivation of youth has been suggested in recent social psychological studies [33]. Significant social relations belonging to university students studying judo-therapy in the Japanese context such as family bonding, friends on the campus, friends off the campus, relations within the classroom, and relations to the community members were carefully encompassed to measure social capital.

Robust multivariable analysis confirmed that social capital measured in relation to bonding and belonging are significantly related to intrinsic motivation and extrinsic motivation positively, and to amotivation negatively (Table 3). The AMS which was applied to measure academic motivation in this study is based on SDT. SDT describes three needs affecting achievement motivation: needs for competency, autonomy, and relatedness [1,6,34]. The components of “relatedness” seem to be influenced by social belonging.

Feeling socially connected to others in an achievement domain would promote a persons’ motivation to succeed in that domain [27,35]. First, when students are academically well-engaged, they can feel a positive self-image from being a good child to their parents, a good student to their teachers and a good friend for their classmates. For example, students who attend the class regularly and study diligently are likely to be regarded by their classmates in a positive way. Their classmates’ appreciation and recognition will then promote the student’s regular attendance in class. Such students can enjoy positive interaction such as sharing knowledge and collaboration in classwork [36]. Moreover, students who are known as good students will be recognized by their family as good children of whom they can be proud. Being embedded in social relationships can lead to a very meaningful social identity and may increase the students’ motivation for academic work [33]. That is why EM and IM of the students are positively influenced by the classroom SC, a measure of bonding and belonging to their teachers and class mates (Table 3). It can be explained further by cultural anthropology.

The cultural value in Japan is high in both group collectivism and institutional collectivism and uncertainty avoidance [9]. In the sociologists’ viewpoint, Japan is a country of high-context culture which is similar to culture in Asian and Middle Eastern countries, but in contrast to America and many European countries where there is low-context culture [37]. Japanese students are more likely to listen than to talk. They are also more likely to accept in the same way as the group rather than asserting their personal opinions. Due to high cultural homogeneity, people always pay attention to intergroup harmony. Students’ feelings of contribution for the good of people and community, teachers’ and peers’ relationship, and sense of belonging to particular society therefore may motivate students. Therefore, “relatedness” in SDT may be the path through which proximal social contexts can influence academic motivation especially in the Japanese and similar Eastern culture [38]. Furthermore, applying the measures of association, our findings can assess which SC is most influential for the student. IM and EM are similarly positively influenced by classroom SC the most, followed by family SC, on-campus friends’ SC, and community SC (Table 3).

Moreover, the findings of the present study can be used to explain the beneficial effect of teaching and learning approaches such as problem-based learning and team-based learning on the learners’ motivation [39,40]. Such small group learning provides an environment for positive interaction among the students themselves, as well as between teachers and students. Those interactions may give rise to good relations among classmates and their feeling of belonging to the class [41]. This may trigger socially-mediated academic motivation of the students and enjoyment in study [27,35]. Hence, the implications of the current study results are diverse. Curricular or extracurricular interventions can be designed based on this finding.

Realizing that social belonging is the source of academic motivation, educators can develop teaching and learning strategies applying socially-driven academic motivation. One example is interprofessional education in which reciprocity, trust, interdependencies and networks acquired through the training of different health professionals together in a team lead to inspiration, academic endeavors and life-long learning. It might nurture students’ academic motivation through good relations on campus [42]. Moreover, there is ample room for future intervention studies using the concept of fostering social capital as a lever for health profession education and universities as contexts for developing social capital. Targeting to enrich the social capital such as early entry to professional society as junior members, or membership in students’ union or web-based forum of the school can be applicable as motivation tools for the students.

The findings in current studies revealed an idea of the classroom as the source of belonging and thus motivation for academic work, in the collectivist society of Japan. Contemporary classroom is essentially a communication community based on belonging and sharing. Teachers are an important source of students’ social capital [43]. The findings encourage the teachers and educators to increase the students’ belonging and connection in university whilst teachers should be aware that education is not just instruction, reward and consequences but that it creates autonomy, competency and relatedness. Furthermore, students learn kindness, compassion, and empathy other than competence of science and clinical skills in the school [44]. It is important to create a classroom in which students are respected, valued and relations with the students are prioritized for their academic motivation and success.

Furthermore, the current study is the first of its kind to investigate the association between social capital and academic motivation in Japan. The strengths of the current study are the use of an adequately powered sample and multi-stage sampling approach as well as validated instruments. Consequently, the generalizability of the results is ensured. Readers should be aware of generalizability to similar population. In addition, the instruments were carefully validated in pilot studies. It is not without limitation. A possible weakness of the study may relate to participants’ social desirability bias upon answering some questions, although they were well informed that their identities would be protected. Furthermore, the association identified in the cross-sectional study is lack of temporality to claim the cause-effect relations. Future longitudinal studies are required. We recruited the data from many schools around the country. In each school, students from different classes and grades were recruited in order to preserve heterogeneity, variance and distribution of the parameters. However, within each class grade, there might be nested nature of the data structure due to homogeneity within each class grade.

## 5. Conclusions

This population level observational study carried out in Japan has affirmed that students’ academic motivation is influenced by students’ individual level social capital. Bonding in the family, belonging to society or class, relation in the community, can foster university students’ motivation for academic work impacting on the affective domain in education. Especially, the development of relations, trust and bonds in the classroom may shape an adult learner’s motivation to evolve into autonomous intrinsic academic motivation and prevent amotivation.

Therefore, the results of this study have opened the way to build social capital-based, new interventions and assemble future research for higher education setting and health profession education, especially in countries with similar cultural context. Furthermore, the result of this study has highlighted the importance within the Japanese social context of enhancing young people’s motivation and interest in the traditionally relayed Japanese health profession of judo-therapy.

## Figures and Tables

**Table 1:** Characteristics of the students in the sample.

Characteristics	N	%
University	925	41.15
College	1323	58.85
Year		
1st	724	32.22
2nd	671	29.86
3rd	614	27.33
4th	238	10.59
Age		
18–19 years	672	29.92
20–29 years	1416	63.05
30–39 years	99	4.41
40–49 years	45	2
50–59 years	11	0.49
>60 years	3	0.13
Gender		
Female	646	28.76
Male	1600	71.24
Work experience		
Yes	301	13.42
No	1942	86.58
Income		
Income of family	1992	90.55
Student’s own income	178	8.09
Other	30	1.36
Scholarship		
Yes	1110	49.62
No	1127	50.38
Academic history of mother		
Junior high school	54	2.51
High school	965	44.93
College	426	19.83
University	703	32.73
Academic history of father		
Junior high school	93	4.44
High school	918	43.82
College	293	13.99
University	791	37.76
Self-determination of course		
Yes	2141	96.18
No	85	3.82
Patient experience		
Yes	2021	90.83
No	204	9.17
Role model		
Yes	1424	63.37
No	823	36.63

**Table 2:** Academic motivation and social capital of students studying judo-therapy, a form of health professional education.

Variables and Constructs	Number	Response Rate (%)	Mean	Standard Deviation	Median	Interquartile Range
**Academic Motivation**						
IM	2247	99.95	54.0	14.2	55	46–63
EM	2247	99.95	54.9	14.1	56	47–64
Amotivation	2247	99.95	10.9	6.1	10	5–16
**Social Capital**						
Family SC	2231	99.24	3.1	0.5	3.1	2.8–3.5
Internal Friend SC	2237	99.51	3.0	0.6	3	2.7–3.5
External Friend SC	2236	99.47	3.0	0.5	3	2.7–3.3
Classroom SC	2223	98.89	2.7	0.5	2.7	2.4–3.1
Community SC	2241	99.69	2.3	0.6	2.3	2.3–2.7

IM—intrinsic motivation; EM—extrinsic motivation; SC—social capital.

**Table 3:** Robust regression analysis of relation between academic motivation (intrinsic motivation, extrinsic motivation and amotivation) and social capital constructs.

	Uni-Variable Regression			Robust Multivariable Regression		
**Intrinsic Motivation**	**β**	**CI**	***p***	**β**	**CI**	***p***
Family SC	0.71	0.60–0.92	**	0.24	0.13–0.35	**
On-campus friends’ SC	1.28	1.12–1.44	**	0.30	0.11–0.50	*
Off-campus friends’ SC	0.55	0.43–0.67	*	0.08	−0.41–0.20	ns
Classroom SC	1.14	1.03–1.24	**	0.73	0.61–0.96	**
Community SC	0.53	0.45–0.61	**	0.23	0.14–0.32	**
Residential formation	1.12	−0.10–2.36	ns	0.60	0.33–2.68	*
Work experience	−2.48	−4.14–−0.81	*	−1.92	−3.44–−0.39	*
Self-chosen career	−7.87	−10.84–−4.90	**	−3.98	−6.66–−1.30	*
Patient experience	−3.51	6.25–8.50	**	−1.42	−3.20–0.35	ns
Role model	7.37	6.25–8.50	*	4.82	3.74–5.90	**
**Extrinsic Motivation**	**β**	**CI**	***p***	**β**	**CI**	***p***
Family SC	0.62	0.51–0.73	*	0.26	0.14–0.38	*
On-campus friends’ SC	1.09	0.93–1.26	*	0.27	0.66–0.47	*
Off-campus friends’ SC	0.58	0.46–0.69	*	0.20	0.07–0.33	*
Classroom SC	0.88	0.78–0.99	*	0.50	0.37–0.63	**
Community SC	0.38	0.30–0.47	*	0.12	−0.03–−0.22	*
Residential formation	1.38	0.14–2.61	*	1.73	0.48–2.97	*
Work experience	0.52	−1.15–2.20	ns	0.48	−1.14–2.10	ns
Self-chosen career	−7.12	−10.11–−4.14	*	−3.16	−6.00–−0.32	*
Patient experience	−3.51	−5.50–−1.53	**	−0.93	−2.82–0.95	ns
Role model	6.56	5.41–7.71	*	4.41	3.27–5.55	**
**Amotivation**	**β**	**CI**	***p***	**β**	**CI**	***p***
Family SC	−0.25	−0.30–−0.20	*	−0.12	−0.17–−0.07	**
On-campus friends’ SC	−0.43	−0.51–−0.36	*	−0.14	−0.23–−0.05	*
Off-campus friends’ SC	−0.09	−0.14–−0.03	**	0.09	0.03–0.15	**
Classroom SC	−0.40	−0.45–−0.36	*	−0.30	−0.35–−0.24	**
Community SC	−0.09	−0.14–−0.06	*	0.03	−0.01–0.07	ns
Residential formation	−0.24	−0.80–0.33	ns	0.35	−0.19–0.89	ns
Work experience	2.54	1.78–3.30	*	2.45	1.74–3.15	**
Self-chosen career	5.31	3.96–6.65	*	3.78	2.54–5.01	**
Patient experience	0.97	0.06–1.88	ns	0.33	−0.48–1.15	ns
Role model	−3.96	−4.47–−3.45	*	−3.12	−3.62–−2.63	**

β—regression coefficient (unstandardized); CI 95%—confidence interval; ***p***—*p*-value; * Significant *p* ≤ 0.05; ** Significant *p* ≤ 0.001; ns *p*-value > 0.05.

## Data Availability

Data is available upon interested researchers’ request, after institutional approval. Please contact corresponding author or the first author.

## Supplementary Materials

The following are available online at https://www.mdpi.com/2254-9625/11/1/11/s1, S1: Social capital scale.
