# Supplementary material for: The Relation between Social Capital and Academic Motivation of Students: A Study of Health Professional Education in Japan
Source: Eur J Investig Health Psychol Educ. 2021 Feb 9;11(1):129–41. doi: 10.3390/ejihpe11010011 (PMC8314329; doi:10.3390/ejihpe11010011)
Supplement: Supplementary file 1 [file ejihpe-11-00011-s001.pdf]

Koyanagi & Aung Social Capital (KASC46) Scale adapted for students in higher education

Koyanagi et.al 2020

Scale Description

To use the scale, please cite "Koyanagi Y, Aung MN, Yuasa M, Sekine M, Takao O. The Relation between Social Capital and Academic Motivation of Students: A Study of Health Professional Education in Japan. *European Journal of Investigation in Health, Psychology and Education*. 2021; 11(1):129-141. https://doi.org/10.3390/ejihpe11010011"

This scale assesses the same 5 constructs as the Social Capital scale toward higher education studies. It contains 46 items assessed on a 4-point scale.

References

Kenji Tsuyuguchi (2016). Relationship between Children's Social Capital and Learning Motivation. Bulletin of Faculty of Education, Ehime University, Vol. 63, 1-12

Adapted by

Yuka Koyanagi ,Myo Nyein Aung,Motoyuki Yuasa,Miwa Sekine,Takao Okada(2020)

Instruction:

We would like to ask you how you are feeling in your everyday life. Please read the following questions, choose the answer that is the closest to how you feel, and circle it.

		Does not apply at all	Does not apply too much	Applies somewhat	Applies extremely well
**No**	**Item**	**1**	**2**	**3**	**4**
1	My family pays close attention to what I say.				
2	Since starting at a university/vocational college, I have not taken part in any activities (meals, sports, travel, etc.) with my family				
3	I actively help my family.				
4	I greet my family ("good morning, good night") and send messages to them.				
5	My family routinely gives me advice about my studies.				
6	I want to make myself useful to my family.				
7	My family cooperates with and helps one another.				
8	My family doesn't help me, even if I'm in trouble.				
9	My family cares about me.				
10	I feel that my family doesn't have high hopes for me.				
11	I take part in activities (meals, sports, travel, etc.) with my school friends.				
12	My school friends don't give me any advice about my studies.				
13	I think I'm helpful to my school friends and people around me.				
14	I always act by cooperating with my school friends.				
15	I feel that my school friends don't rely on me.				
16	When I experience stress, my school friends help ease it.				
17	I have friends outside the campus, such as at my parttime job and sports clubs.				

No	Item	Does not apply at all<br>1	Does not apply too much<br>2	Applies somewhat<br>3	Applies extremely well<br>4
18	I keep in touch with my classmates from elementary, middle and high schools.				
19	I have people who are my friends on SNS only.				
20	I take part in activities (meals, sports, travel, etc.) together with my friends outside the campus.				
21	My friends from outside the campus give me advice about my studies.				
22	I think I'm helpful to my friends from outside the campus, and to the people around me.				
23	I always act by cooperating with my friends from outside the campus.				
24	I feel that my friends from outside the campus don't rely on me.				
25	I can feel secure when I'm with my friends from outside the campus.				
26	My teachers (seminar supervisors, class teachers, advisors, etc.) listen closely to what I say.				
27	I actively belong to sports clubs and group activities.				
28	I take an active part in cultural festivals and other school events.				
29	In my class, I take the initiative in greeting my classmates.				
30	My teachers (seminar supervisors, class teachers, advisors, etc.) give me advice about my studies.				
31	I want to make myself useful to the people in my class.				
32	I feel that the people in my class are not helping each other.				
33	In my class, if I'm in trouble, people around me offer help.				
34	My teachers (seminar supervisors, class teachers, advisors, etc.) care very much about me.				
35	I have people who are my friends on SNS only.				
36	I feel that my teachers don't have high hopes for me.				
37	I take part in events carried out in the neighbourhood where I live.				

No	Item	1 Does not apply at all	2 Does not apply too much	3 Applies somewhat	4 Applies extremely well
38	I say hello to the people in my neighbourhood where I live.				
39	I take part in sports and cultural groups in the area I currently live in.				
40	I want to carry out activities in the community and at school, with the residents in the area I currently live in.				
41	I take part in volunteer activities in the area I currently live in.				
42	I want to make the area I currently live in, an even better area.				
43	I want to become able to contribute to the area I currently live in.				
44	I feel that I cannot trust the residents of the area I currently live in.				
45	I like the area I currently live in.				
46	I don't want to continue living forever in the area I currently live in.				

Note: Original Japanese version instrument was used to administer to the participants in the research. This is English version instrument, translated to English by professional translator for the purpose of peer review, publication and sharing instrument to future researchers.

Key for the calculation of score

1,2,3,4,5,6,7,8,9,10 reverse item:2,8,10	Family social capital
11,12,13,14,15,16 reverse item:12,15	On-Campus friends social capital
17,18,19,20,21,22,23,24,25 reverse item:24	Off-Campus friends social capital
26,27,28,29,30,31,32,33,34,35,36 reverse item:32,35	Classroom social capital
37,38,39,40,41,42,43,45,46 reverse item:46	Community social capital
